# Alteration of the foot center of pressure trajectory by an unstable shoe design

**DOI:** 10.1186/s13047-015-0124-3

**Published:** 2015-12-01

**Authors:** Mona Khoury, Amir Haim, Amir Herman, Nimrod Rozen, Alon Wolf

**Affiliations:** Biorobotics and Biomechanics Lab (BRML), Faculty of Mechanical Engineering, Technion-Israel Institute of Technology, Haifa, 32000 Israel; Division of Orthopedics, Sourasky Medical Center, Tel Aviv, Israel; Department of Orthopedics and Talpiot Medical Leadership Program, Sheba Medical Center, Tel Aviv, Israel; Department of Orthopaedic Surgery, Ha’Emek Medical Center, Afula, Israel

**Keywords:** Gait, Foot center of pressure, Plantar pressures, Unstable shoe design

## Abstract

**Background:**

Unstable sole designs have been used as functional or therapeutic tools for improving body stability during locomotion. It has been suggested that the narrow base of support under the feet generate perturbations that challenge the instability of different joints during motion, thereby forcing the body to modify its movement in order to maintain a stable gait. The purpose of the present study was to explore the correlation between the stability of the footwear-device and the magnitude of perturbation conveyed during gait.

**Methods:**

Various levels of dynamic instability were achieved using a novel foot-worn platform with two adjustable convex rubber elements attached to its sole. A total of 20 healthy male adults underwent direct in-shoe pressure measurements while walking with the footwear device. Foot center of pressure (COP) and stride to stride variability measures were extracted to examine the correlation between the magnitude of the instability and the imposed perturbations during gait.

**Results:**

A counterintuitive but significant correlation was found between stride to stride variability and the instability of the biomechanical elements. Moreover, there was significant correlation between the instability of the elements and the perturbations found in the COP trajectory. The linear model describing this correlation was found to be statistically significant.

**Conclusion:**

There was significantly negative correlation between the level of instability induced by the shoe design and the amount of perturbations conveyed during gait. This suggests that the external perturbation must remain within a certain range limit. Exceeding this limit can negatively affect the treatment and probably lead to opposite results.

## Background

In recent years, a novel therapeutic approach to musculoskeletal pathologies, centered on neuromuscular reeducation, has emerged and has been the focus of a vast amount of research [1–3]. The principal behind these therapeutic interventions is that the neurological system controlling locomotion is plastic and, given accurate stimuli, can generate enhanced motor activation patterns that can compensate for anatomical pathologies which compromise gait [4].

It has been suggested that perturbation can generate the appropriate stimuli to improve proprioception and to adopt altered motor control strategies during gait. Footwear-generated biomechanical manipulations have been commonly utilized for this objective [5]. Acting as an interface between the feet and the ground, footwear can manipulate sensory feedback information originating from the plantar surface of the foot and generate these stimuli. The idea behind these designs is to introduce controlled diminished support, thereby challenging joint stability and balance control, a strategy that may allow users to develop motor skills adequate to protect their joints from potentially harmful loads during functional activities [6].

Several unsteady shoe designs have been developed and have been reported to produce favorable outcomes of functional activity and pain reduction [6–10]. Findings related to the effect of the MBT unstable shoe sole showed that wearing these shoes in a standing position increases the movement amount of the trajectory of the center of pressure (COP) [3]. Erhat et al. reported that training with the partial stiffness sole shoe in patients suffering from knee osteoarthritis (OA) resulted in decreased knee adductor moment [8]. Recent studies by our group have focused on a novel foot-worn biomechanical device (APOS therapy) which consists of a foot-worn platform with two convex-shaped rubber elements attached to its sole. The elements are available in different levels of convexity and can be shifted and rotated in any position under the sole of the shoe to allow various biomechanical manipulations. We found that controlled shifts of the elements can significantly alter the location of the foot COP [11], thereby influencing kinetic and kinematic parameters of gait for both healthy individuals and OA patients [12]. Moreover, the convex nature of the elements gives the device slight unsteadiness when worn and, since subjects can wear the device while walking, the device induces dynamic perturbations during walking. It was suggested that repetitive and programmed use of the device may lead to motor learning and gait corrections in patients suffering from knee osteoarthritis (OA) [1, 2, 5, 11, 12].

However, the effect of the instability of the elements on the level of perturbation generated has yet to be established. It is well accepted that perturbation manifests as stride to stride variation of gait parameters as well as in the trajectory of the foot’s COP [11]. Therefore, we devised the current study to explore the association between the stability of the foot-device and the magnitude of perturbation conveyed during gait. We hypothesized that increasing the instability would be reflected in stride to stride gait variability (STSV) as well as in the magnitude of fluctuations of the COP trajectory.

## Methods

### Participants

The study cohort was comprised of 20 healthy male volunteers without any known history of injury or any postural or skeletal disorder which could affect normal posture or gait. All participants had an equivalent shoe size (French 43), a right dominant leg and a similar anthropometric profile. The participants’ mean ± SD age was 24 years ± 11 months, height was 176.1 ± 3.29 cm, and body mass was 68.8 ± 6.67 kg. Approval of the local Ethics Committee Ha'Emek Medical Center (Afula-Israel) was obtained and informed consent was given by all participants.

### The biomechanical system

**Fig. 1:**
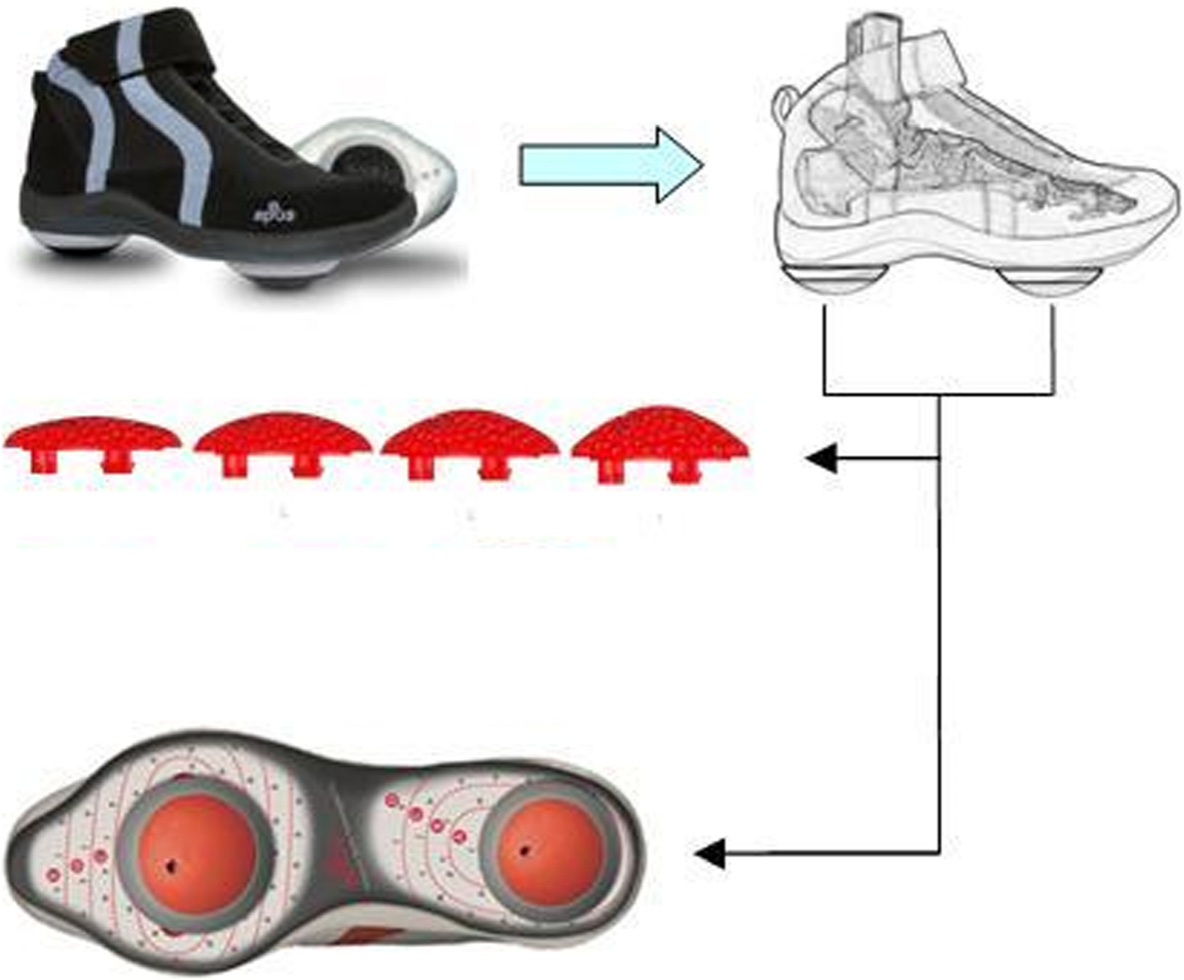
Biomechanical platform and the rubber elements with four different convexities

The biomechanical device (APOS-Medical and Sports Technologies Ltd., Herzliya, Israel) utilized in this study has been previously described in detail [11]. In brief, the device comprises a foot-worn platform with two adjustable convex rubber elements attached to its base, one under the hind foot and one under the forefoot (Fig. 1). The convex nature of the elements gives the device a slight unsteadiness when worn. The convexity of the elements increases as the height of the elements increases. A variety of elements of different heights can be attached and interchanged on the device. For the purposes of the current study we utilized four different heights. Each height defined different level of stability. Elements with a height of 7.3, 9.2, 10.8 and 12.2 mm defined Level 1, 2, 3 and 4 accordingly. Were level 1 was defined as the lowest instability and level 4 the highest instability.

### Experimental protocol

Prior to testing, participants were functionally assessed by a physiotherapist and were asked to walk several minutes with the shoe-device, in order to become accustomed to it. Then, through observations of the subject’s walk and his feedback regarding the comfort of use during walking, a qualified physiotherapist defined the “functional neutral configuration” (FNC) to which the elements were fixed throughout the whole experiment. For healthy users, FNC was defined as the position of the elements in which the apparatus exerts the least valgus, varus, dorsal or plantar torque about the ankle. This procedure has been previously described [11, 13]. Four successive plantar pressure testing were performed, each with a different stage of instability as defined earlier. The only difference between the testing conditions was the convexity of the elements.

All measurements were applied while the participant walked over a flat 30 m long surface, at a constant self-selected velocity. At the beginning of each experiment we have captured the typical walking speed for each participant and then fixed a metronome to that rhythm so that the walking velocity remains constant during all experiment conditions. All conditions were tested in arbitrary order on the same day. To insure uniformity of testing conditions, all subjects were provided with the same biomechanical system and all calibrations of the biomechanical device were performed by the same physiotherapist.

### Data acquisition and processing

Plantar pressure testing was applied using the Pedar-x Pressure-measurement system (Novel Electronics, St. Paul MN, USA) which consists of 2.5 mm thickness insoles incorporating a matrix of 99 pressure-sensitive capacitive insoles which can be placed into the subject’s footwear to measure pressure during gait. The sensors were sampled at a rate of 100 Hz. Foot COP data was extracted and analyzed for the dominant leg using Matlab.

Stride to stride variability (STSV) was defined for each of the testing condition as standard deviation in stance length for each testing condition and directly extracted from the Pedar system.

Moreover, we defined two measures to examine if the level of instability was reflected in the foot COP trajectory. The first measure was the perturbations of the COP data and was calculated as the root mean square change in medial-lateral position of the COP during each stance phase. Mean values were calculated for each level of instability as the average value of all steps in the same trial. The second measure was defined as the mean medio-lateral location of the COP trajectory and was evaluated for four sub-phases of the stance phase: loading response (LR): 0 to 10 %; mid-stance (MS): 10 to 30 %; terminal stance (TS): 30 to 50 %; and pre-swing (PS): 50 to 60 %. Values were averaged over all steps.

To determine if the stage of instability affected STSV and both COP measures, three linear mixed effect models were fitted. In one model, STSV was the dependent covariate with element height as fixed effect factor and patient’s effect as the random effect. The second linear model included COP perturbations as the dependent covariate and element height as fixed effect with patient’s effect as random factor. In the third model, the mean COP location was the dependent covariate with element height and gait sub-phase as fixed effect factors and patient’s effect as the random effect. A *p*-value of less than 0.05 was considered to be statistically significant. All pvalues reported are two sided.

## Results

The first linear model describing STSV as depending on the level of instability is *STSV* = 2.45 − 0.055 × *Height* which means that for every mm of element height there was 0.055 mm less variability in the stance length. This is statistically significant (*p* = 0.048).

The linear model for COP perturbations as depending on the level of instability is *Pert* = 0.44 − 0.005 × *Height*. The slope is statistically significant (*p* = 0.005). This means that for every mm of biomechanical element height the perturbations decreases by 0.005 mm.

**Fig. 2:**
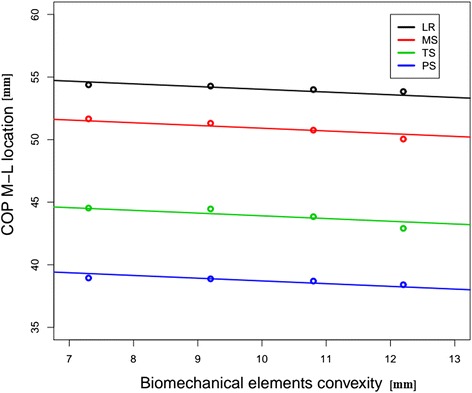
Mean COP M-L location vs biomechanical elements height, according to gait stage. The points represent mean COP for each element (height) at each gait stage. The linear models show that as the height increases (level of instability), the COP moves medially. This effect is similar for every gait stage. *COP* center of pressure, *M*-*L* mediolateral, *LR* loading response, *MS* mid-stance, *TS* terminal stance, *PS* preswing

Mean values and standard deviations of the mean COP measured with four different levels of convexity are presented in Table 1. The COP trajectory throughout the stance shifted medially as the stage of instability induced by the biomechanical element was increased. A linear model for the shift as a function of element height and gait stage was *COP* = *A* + *B* × *Conv*. The intercept (A) changed with gait stage being 56.28, 53.09, 46.09 and 40.89 mm for stages LR, MS, TS and PS, respectively. The Slope was −0.21 mm (±0.09) for each mm of height of the biomechanical elements. This means that for each mm of height the COP shifted medially by 0.21 mm. This was found to be statistically significant (*p* = 0.001). The model fit the data well (${R}^{{}^2}$ = 0.796, overall model *p* = 0.001). In all the gait stages, the effect of the stage of instability on COP position was similar, i.e. the interaction terms were not statistically significant and were excluded from the model. The linear model for each gait stage and the COP position are presented in Fig. 2.

**Table 1:** Mean ± std. dev values of the medio-lateral COP in mm (*n* = 20) at different levels of instability (height of different elements)

	Stance sub-phase							
Element height	Loading response		Midstance		Terminal stance		Preswing	
7.3 mm	54.35	±1.88	51.5	±3.05	44.33	±3.71	38.61	±3.74
9.2 mm	54.26	±2.45	51.23	±3.42	44.36	±3.52	38.53	±3.35
10.8 mm	54	±2.21	50.57	±2.66	43.56	±2.86	38.25	±3.09
12.2 mm	53.82	±2.37	49.95	±2.92	42.72	±3.02	38.03	±3.32

## Discussion

Unsteady sole designs have become increasingly popular as both therapeutic and functional tools. The goal of this design is to generate perturbation during gait. The results of the current study offer a quantitative correlation between the convex design of the sole of the shoe and the magnitude of perturbation conveyed. In contrast to the study hypothesis, a negative association was found between the height of the biomechanical element and the perturbations induced by the shoe. Our results showed that, with increasing the height of the elements by 1 mm, there was 0.055 mm less stance to stance variability, and 0.005 mm *less* perturbations in the foot COP.

Analysis of the COP locus, on the other hand, demonstrated that, with the same increase in the convexity of the elements, a 0.21 mm medial shift in the COP trajectory occurred. We speculate that this change of trajectory location may explain the above results and indicate that coping with increased unsteadiness occurs via inversion of the ankle joint, which results in a medial COP shift and diminished perturbations.

A similar finding was reported by Han et al. who investigated the effect of high-heeled shoes on planter pressure data and found that the COP locus was inwardly (medially) displaced during the stance phase of high-heeled gait compared to flat heeled gait [14]. They also found that heel height reduces the contact area of the total plantar surface and the contact width of the mid-foot. It may be arguable whether assumptions concerning ankle joint motion can be made from observations of the COP patterns alone or not. However, it is self evident that pronation of the foot increases the total contact area. If the use of high heels reduces the plantar surface area, it can be inferred that the foot becomes more plantar flexed and supinated. This supports our assumption that an increased level of instability emboldens inversion of the subtalar joint. Future studies incorporating ankle joint kinematics and kinetics together with COP examinations could provide more validity to these speculations [4].

It should be noted that the velocity was set to the normal walking pace of each patient and was constant during the whole experiment in all four testing conditions, so that the effect of velocity on COP and spatiotemporal measures can be excluded.

Putting the results together, we conclude that increasing the instability of the shoe results in less perturbations in the COP trajectory, less stride to stride variability, and a medially locked COP. If it is correct to assume that higher convexity causes more instability when worn during gait then, our speculation is that, at some point, the external manipulation becomes extreme so that, in order to maintain stable and safe progression, the body is forced to make modifications that result in a mechanical stop of the ankle joint at the eversion position. Increased activity of the foot evertors takes the foot away from its medial plane and results in a medially displaced COP [15].

Attending to the outcomes of this study, external foot-based manipulations should be used in moderation. Extreme steep designs can lead [16] to increased reactions to maintain stability during gait and, therefore, might negatively affect the treatment. Investigating smaller convexities, although not available by the system we used for this research, might have given the opposite and more intuitive result.

Reduced ability to control balance during gait (e.g., older individuals) is expected to affect these relations and, therefore, should be further studied. Moreover, Nigg et al.investigated gender differences in lower extremity gait kinematics and kinetics when using unstable shoes and reported that gender effects should be taken into consideration if functional or therapeutic effects of unstable shoes are assessed. Their results suggest that women and men use different strategies to control the ankle joint when walking in unstable shoes and, therefore, are expected to affect COP patterns [16].

It should be noted that the results of the current study are valid only for individuals with characteristics similar to those of the study cohort (i.e. healthy, young male adults). Further studies are required to validate these findings in other populations.

It should also be noted that the biomechanical testing was performed shortly after the device was first used by the participants. Continued usage of such an apparatus may lead to substantial gait adaptations and could influence the outcome of these interventions.

Finally, it should be noted that the apparatus was employed at neutral position as a base condition for the experiment. This was set by a trained physiotherapist who watched the patients walk and moved the elements untill a minimum varus/valgus moment was achieved. We are aware of the limitations of this method, however, it has been previously used [5]. The position of the FNC remained constant during the whole experiment. We only changed the elements convexity and compared the differences so that each patient was a control of himself. Moreover, there was no control condition. All conditions included the convex elements from the least to the most convex. This is because the sole of the shoe is too rigid to be used without the elements and therefore could not function as control.

Future studies considering the interaction effect between the convexity and the location of these elements are needed for better understanding of the foot COP and its relation to the apparatus utilized. Another limitation of the apparatus shoe design which should be pointed out, is that it provides ankle stability that could affect the motion and the joints and alter our results. Nevertheless, the current study is unique in that it offers a quantitative correlation between the instability (height) of the elements and the amount of perturbation produced during gait and brings a whole new aspect for the use of unstable therapeutic devices that can be extremely important for practitioners.

## Conclusions

The current study examines the correlation between the instability of the shoe design and the amount of perturbations produced during gait.Understanding the effect of the shoe instability on the generated perturbatios is cruitial for understanding the strategy of utilizing such devices and the expected compliance of users. The findings of this research could therefore, have great implications on the apparatus device and practice.
